# Patterns of respectful care and mistreatment during childbirth in relation to perinatal mental health: a secondary analysis of listening to mothers in California

**DOI:** 10.1007/s00737-025-01656-0

**Published:** 2026-03-07

**Authors:** Stacey Iobst, Elise Erickson

**Affiliations:** 1 Department of Nursing, Towson University, Maryland Towson, United States; 2 Department of Physiology, The University of Arizona, Arizona Tucson, United States

**Keywords:** Anxiety, Depression, Maternity care, Mistreatment, Respect

## Abstract

**Purpose:**

Despite mental health conditions being the leading cause of maternal mortality in the United States, the relationship between perinatal mental health and mistreatment during childbirth has been insufficiently examined. The objective was to identify patterns of respectful care and mistreatment during childbirth and examine associations with perinatal mental health.

**Methods:**

We conducted a cross-sectional secondary analysis of Listening to Mothers in California (*N* = 2,539). We conducted latent class analysis of indicator variables describing rough or rude care, discriminatory treatment, support, communication, and encouragement of autonomous decision-making. We conducted multinomial logistic regression to examine symptomatology for antenatal anxiety and antenatal depression in relation to class membership. Logistic regression was used to examine class membership in relation to symptomatology for postpartum anxiety and postpartum depression.

**Results:**

Four latent classes were identified: Class 1 (84.54%): Respected/Supported, Class 2 (6.69%): Not Supported, Class 3 (5.39%): Rough/Rude, Class 4 (3.38%): Rough/Rude/Discriminated. Women with antenatal depression symptomatology were 3.28 times as likely to be classified in Class 4 versus Class 1 (aRRR 3.28, CI95% 1.88–5.73). Women with antenatal anxiety symptomatology had a higher risk of classification in Class 4 than Class 1 (aRRR 2.04, CI95% 1.21–3.43). Compared to women in Class 1, women in Class 2 had 2.06 higher adjusted risk of postpartum anxiety (aRRR 2.06, CI95% 1.26–3.36).

**Conclusions:**

Women who were symptomatic for antenatal anxiety or antenatal depression were at increased risk for mistreatment and not receiving support during childbirth. Women who did not feel supported had increased risk of symptomatology for postpartum anxiety.

**Supplementary Information:**

The online version contains supplementary material available at 10.1007/s00737-025-01656-0.

## Article highlights

Four latent classes of respectful care and mistreatment during childbirth were identified.

Symptoms of antenatal anxiety or antenatal depression were associated with increased risk for rough, rude, and discriminatory treatment.

Not feeling supported during childbirth was associated with increased risk of symptomatology for postpartum anxiety.

**Supplementary Information:**

The online version contains supplementary material available at 10.1007/s00737-025-01656-0.

## Introduction

Mental health conditions are the leading cause of maternal mortality, contributing to 22.7% of deaths in the United States (U.S.) (Trost et al. 2022). Most research to date has focused on the occurrence of anxiety and depression during the postpartum period despite these conditions also affecting women antenatally during pregnancy. This approach overlooks the importance of antenatal anxiety and depression as risk factors for adverse birth experiences, including mistreatment during childbirth, which has received increased global attention in recent years.

Mistreatment during maternity care has been reported by 13% to 20% of women in the U.S. (Liu et al. 2024; Mohamoud et al. 2023; Vedam et al. 2019) and has gained increased attention due to an association with maternal mortality (Indiana Department of Health 2022; New York State Department of Health Maternal Mortality Review Board 2020). The most frequent types of mistreatment in U.S. maternity care include neglect and being shouted at or scolded (Liu et al. 2024; Vedam et al. 2019). Women also report lack of support, with nearly half refraining from asking questions or discussing concerns with providers (Mohamoud et al. 2023).

Critically, the relationship between perinatal mental health and mistreatment during pregnancy is poorly understood. Few studies have examined antenatal anxiety or antenatal depression as risk factors for mistreatment during maternity care and results to date have been mixed (Gürber et al. 2017; Liu et al. 2024). Moreover, evidence has indicated an association between perception of a negative birth experience and risk for postpartum depression (Basile-Ibrahim et al., 2024; Bell and Andersson 2016; Coo et al. 2023; Gürber et al. 2017) and postpartum anxiety (Basile-Ibrahim et al., 2024; Coo et al. 2023). However, specific types of mistreatment have been examined to a lesser extent in relation to postpartum mental health (Guure et al. 2023; Weeks et al. 2022).

To address gaps in the evidence, our study aimed to (1) identify patterns of respectful care and mistreatment during childbirth, (2) examine antenatal anxiety and antenatal depression as risk factors for mistreatment during childbirth, (3) and examine postpartum anxiety or postpartum depression as risk factors for mistreatment during childbirth.

## Methods

We conducted a cross-sectional, secondary analysis of the Listening to Mothers in California (LTMCA) survey conducted in 2017. Participants were eligible if they were at least 18 years old, gave birth to a single infant in a hospital in 2016, were living with the infant when the survey was administered, and could respond to the survey in English or Spanish. A weighting variable was created to make the sample representative of the women who gave birth in California in 2016. Additional details are reported by Sakala et al. (2018) The de-identified data are publicly available. The study was determined to be not human subjects research by the Towson University institutional review board (protocol #1392).

### Variables of interest

#### Perinatal mental health

Symptoms of anxiety and depression during the antenatal and postpartum periods were measured using the Patient Health Questionnaire (PHQ-4). The PHQ-4 consists of two items measuring symptoms of depression via the Patient Health Questionnaire (PHQ-2) and two items measuring anxiety symptoms via the Generalized Anxiety Disorder questionnaire (GAD-2). In studies using the PHQ-4, the Cronbach’s alpha ranged from 0.65 to 0.81 for the depression (PHQ-2) subscale and from 0.74 to 0.84 for the anxiety (GAD-2) subscale (Caro-Fuentes and Sanabria-Mazo 2024). In the LTMCA survey each item was scored using a four-point Likert scale (1 = never, 2 = sometimes, 3 = usually, 4 = always). To reflect scoring of the PHQ-4 as originally developed by Kroenke et al. (2009), we recoded the Likert scale from 0 to 4. We then summed scores for the PHQ-2 and GAD-2 items, respectively, and coded a total score of ≥ 3 as “yes” and < 3 as “no” for being symptomatic for depression or anxiety, which is consistent with score interpretation by the developers of the instrument (Kroenke et al. 2009) and in other maternity care research examining the Listening to Mothers dataset (Feinberg et al. 2022). The following eight indicator variables were used in our latent class analysis (LCA):

#### Rough/rude treatment

Two survey questions examined rough or rude care: “During your recent hospital stay when you had your baby did a nurse or maternity care provider (1) ever handle you roughly? or (2) ever use harsh, rude, or threatening language?” Responses were coded as 1 = yes and 2 = no. We recoded these variables so that responses were coded as 1 = yes and 0 = no.

#### Discriminatory treatment

Three survey questions examined discriminatory treatment: “During your recent hospital stay when you had your baby how often were you treated unfairly because of (1) your race or ethnicity, (2) the language you spoke, and (3) the type of health insurance you had or because you didn’t have health insurance.” Responses were coded using a four-point Likert scale (1 = never, 2 = sometimes, 3 = usually, 4 = always). We collapsed variables so that “never” = 0 (no) and “sometimes”, “usually”, or “always” = 1 (yes).

#### Support and communication

Three survey questions examined support and communication: “How much do you agree with the following statements about your recent experience of labor and birth? (1) The delivery room staff encouraged me to make decisions about how I wanted my birth to progress, (2) I felt well supported by staff during my labor and birth, and (3) The staff communicated well with me during labor.” Responses were coded using a five-point Likert scale (1 = agree strongly, 2 = agree somewhat, 3 = neither agree nor disagree, 4 = disagree somewhat, 5 = disagree strongly). We collapsed variables so that “agree strongly” or “agree somewhat” = 2 (agree), “neither agree nor disagree” = 1 (neutral), and “disagree somewhat” or “disagree strongly” = 0 (disagree).

### Analytic approach

Class of care, determined by LCA indicators, was the independent variable. We conducted LCA in Latent GOLD ^®^ 6.0. A series of models were tested by fitting 2 to 7 class solutions with maximum likelihood estimation method and missing values retained. We examined model differences by comparing fit statistics. The best classification is typically identified by the model with the lowest Akaike information criterion (AIC) and Bayesian information criterion (BIC). The p-value associated with the Vuong-Lo-Mendell-Rubin (VLMR) adjusted likelihood ratio test is used to select the best fitting model, where the next lower-class model is rejected when *p* < 0.05 (Lo et al. 2001; Vuong 1989). Bivariate residuals indicate whether the local independence assumption is violated, where bivariate residuals less than 3 are desired. There is no consensus about cutoff for entropy; however, a value closer to 1.0 is considered ideal (Gilles 1996).

In addition to these fit statistics, we considered sample size of each latent class (i.e., ideally no less than 50) (Muthén and Muthén 2000) and theoretical interpretability when selecting a model. We did not include the sample weight in LCA because an unweighted LCA is the preferred approach when variables used to construct sampling weights do not affect measurement of the latent class model (Vermunt and Magidson 2007).

For LCA, a sample size of 300 or more is considered adequate (Nylund-Gibson and Choi 2018). We estimated required sample size for logistic regression using G*Power 3.1. Assuming a risk ratio of 0.5 to represent a small effect size, the minimum sample size needed to achieve an alpha level of 0.05 with a power of 0.95 was estimated to be 505 respondents.

**Fig. 1:**
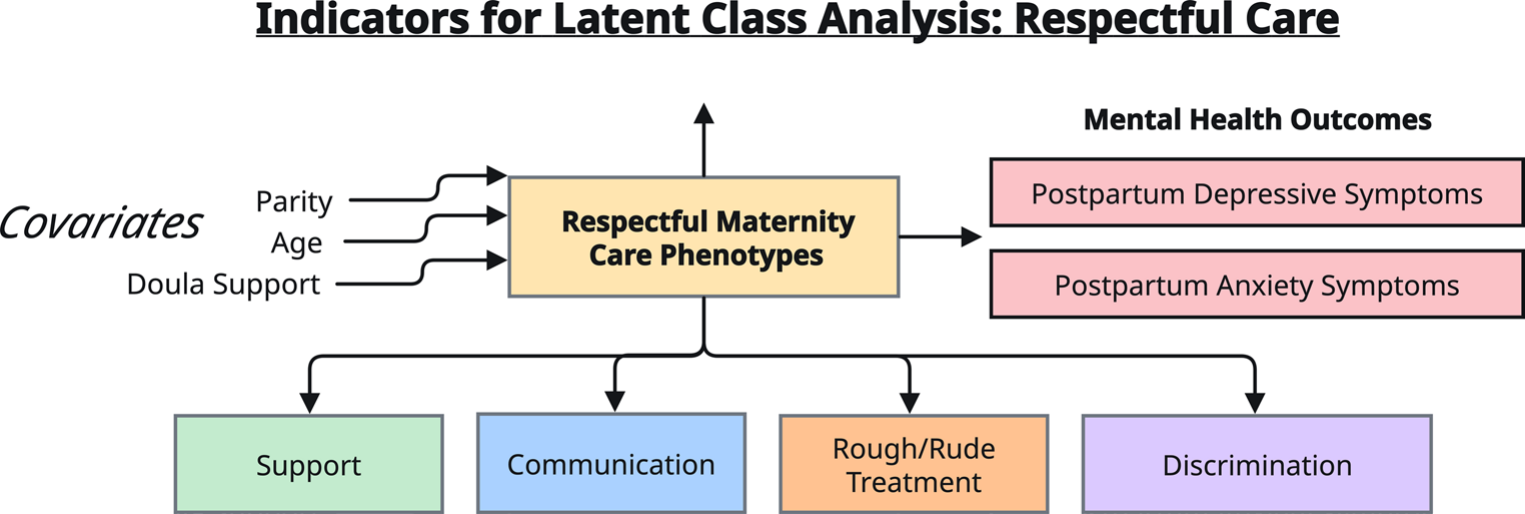
Directed acyclic graph of indicators for latent class analysis, mental health outcomes, and covariates

Descriptive statistics were calculated for the overall sample and for each identified latent class. Differences among classes in relation to demographic and clinical variables were examined using adjusted Wald and chi-squared tests. We conducted multinomial logistic regression to examine associations between having symptoms of antenatal anxiety (GAD-2 ≥ 3) or antenatal depression (PHQ-2 ≥ 3) and class membership. Then we used logistic regression to examine if class membership increased risk for symptoms of postpartum anxiety (GAD-2 ≥ 3) or postpartum depression (PHQ-2 ≥ 3). To identify covariates for inclusion in our regression models, we created a directed acyclic graph to identify each variable’s position within the causal pathway. Maternal age, parity, and presence of a doula were identified as covariates for inclusion in multinomial logistic regression models (Fig. 1). We also included being symptomatic for antenatal anxiety or antenatal depression as covariates in logistic regression models based on evidence of these conditions as risk factors for postpartum anxiety or postpartum depression (Hutchens and Kearney 2020).

Data preparation, descriptive statistics, and regression modeling were conducted using Stata version 16.1 (College Station, TX). We used the sample weighting variable in descriptive and regression analyses.

## Results

### Sample selection

A total of 2,539 respondents completed the LTMCA survey (Table 1). In the overall sample, weighted to represent births in California in 2016, mean age was 29.61 years (SD 0.12). Most were married (86.15%), had some college education (32.97%), and had Medi-Cal public insurance through the state of California (48.62%). Although half of respondents were Hispanic (50.07%), more than half (57.07%) reported speaking English at home. Mean parity was 2.06 (SD 0.03) and mean gestation was 38.80 (SD 0.04) weeks. Only 16.37% of respondents had a doula present at birth. Most had a choice about their prenatal care provider (79.96%) and had an obstetrician/gynecologist for prenatal care (80.29%) and at delivery (72.51%).

**Fig. 2:**
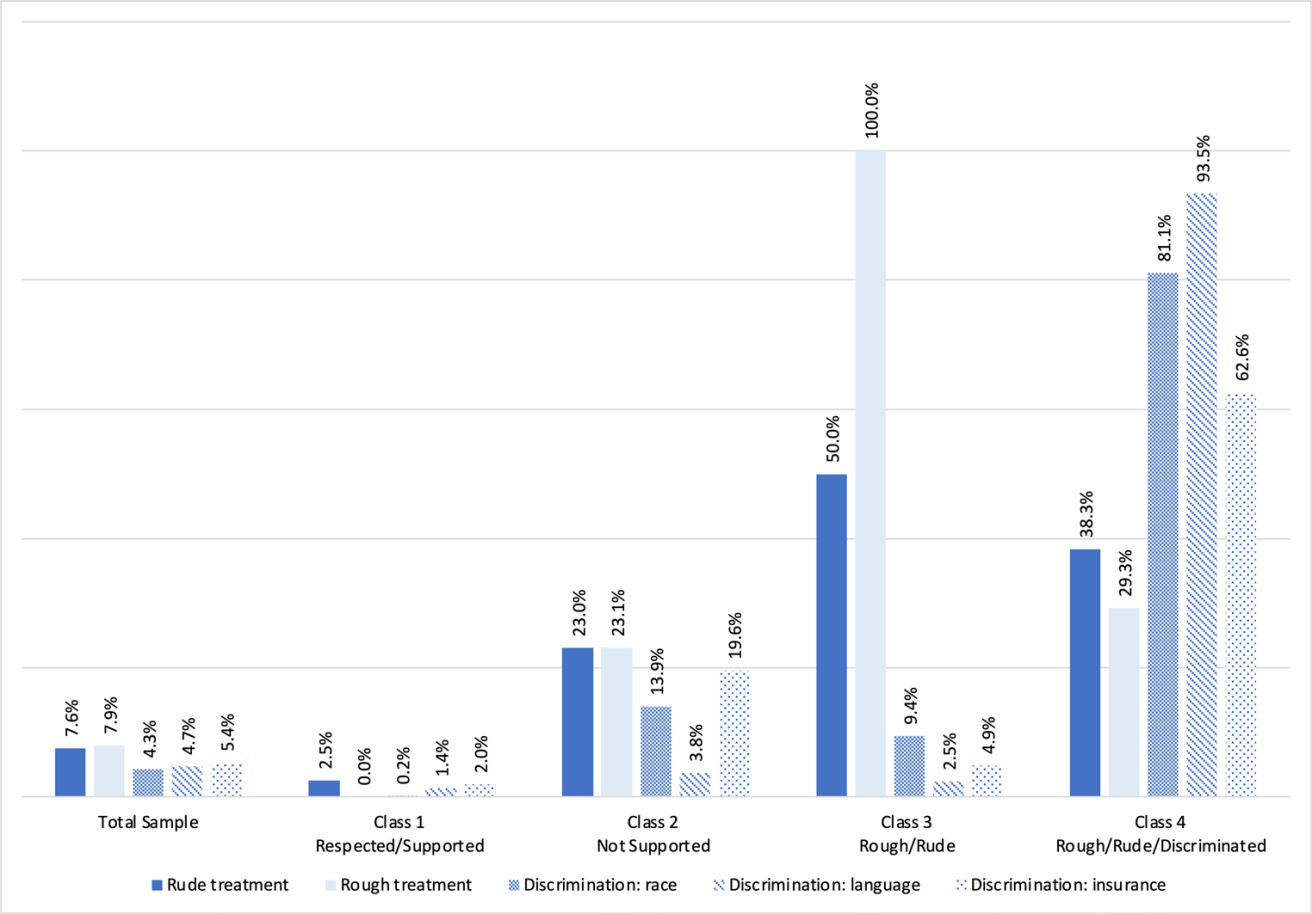
Percentages of respondents reporting types of mistreatment by class membership

**Fig. 3:**
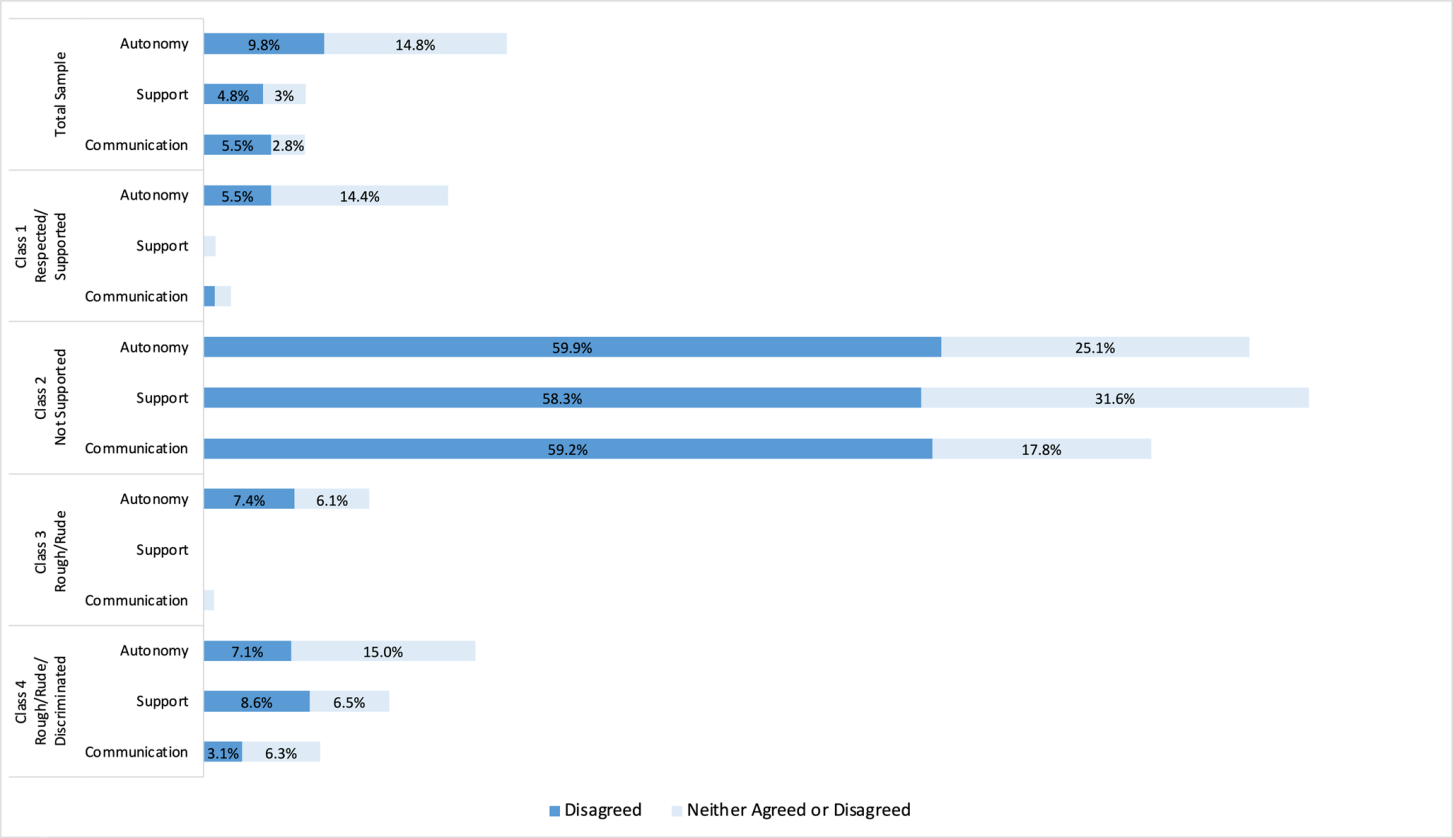
Percentages of respondents reporting not receiving types of support by class membership

In the whole sample, mistreatment was identified by 4.3% of participants reporting discrimination based on race and 7.9% reporting rough treatment by a nurse or maternity care provider (Fig. 2). Overall, 7.8% did not feel well-supported by staff during labor and birth, 8.3% did not have good communication with staff, and 24.6% did not feel encouraged to make decisions about their birth (Fig. 3).

We identified a 4-class model as the best classification of care (See supplemental file 1). To assist with interpretation we labeled classes by whether respondents reported being respected and/or supported.

#### Class 1: Respected/supported

This was the largest class (*n* = 2146 unweighted, 84.54% weighted). Only 3% of respondents reported experiencing mistreatment across all types. Only 0.96% were neutral about feeling well-supported and 2.2% were neutral or denied having good communication with staff. A larger percentage of respondents (19.9%) denied feeling supported in autonomous decision-making.

#### Class 2: Not supported

This class was the second largest (*n* = 176 unweighted, 6.69% weighted). Most respondents ( 58.3%) denied feeling well-supported, 59.2% denied having good communication with staff, and 59.9% denied feeling supported in autonomous decision-making. The percentage of English-speaking respondents (64.65%) in Class 2 was higher than any other class or the overall sample.

#### Class 3: Rough/rude

All respondents in class 3 (*n* = 131 unweighted, 5.39% weighted) reported experiencing rough treatment from a nurse or maternity care provider and 50.0% reported experiencing rude treatment from a nurse or maternity care provider. Interestingly, all members of Class 3 also reported experiencing supportive care (100.0%) and nearly all reported good communication (99.2%). The majority reported being encouraged to conduct autonomous decision-making (86.5%). Reports of discriminatory treatment were relatively low. Members of Class 3 were the most highly educated and had the highest proportion of respondents with private health insurance compared to other classes.

#### Class 4: Rough/rude/discriminated

This was the smallest Class (n = 86 unweighted, 3.38% weighted) and had a high proportion of respondents who reported discrimination based on language spoken at home (93.5%), race (81.1%), and insurance status (62.6%). Class 4 had relatively high proportions of women reporting rude (38.3%) or rough treatment (29.3%) from a nurse or maternity care provider. Class 4 had the highest proportion of Hispanic/Latina (56.08%) and Asian/Pacific Islander (33.32%) respondents, Spanish-speaking only respondents (35.83%), and respondents with Medi-Cal public health insurance (74.79%).

### Antenatal mental health symptoms were more prevalent than postpartum symptoms

**Fig. 4:**
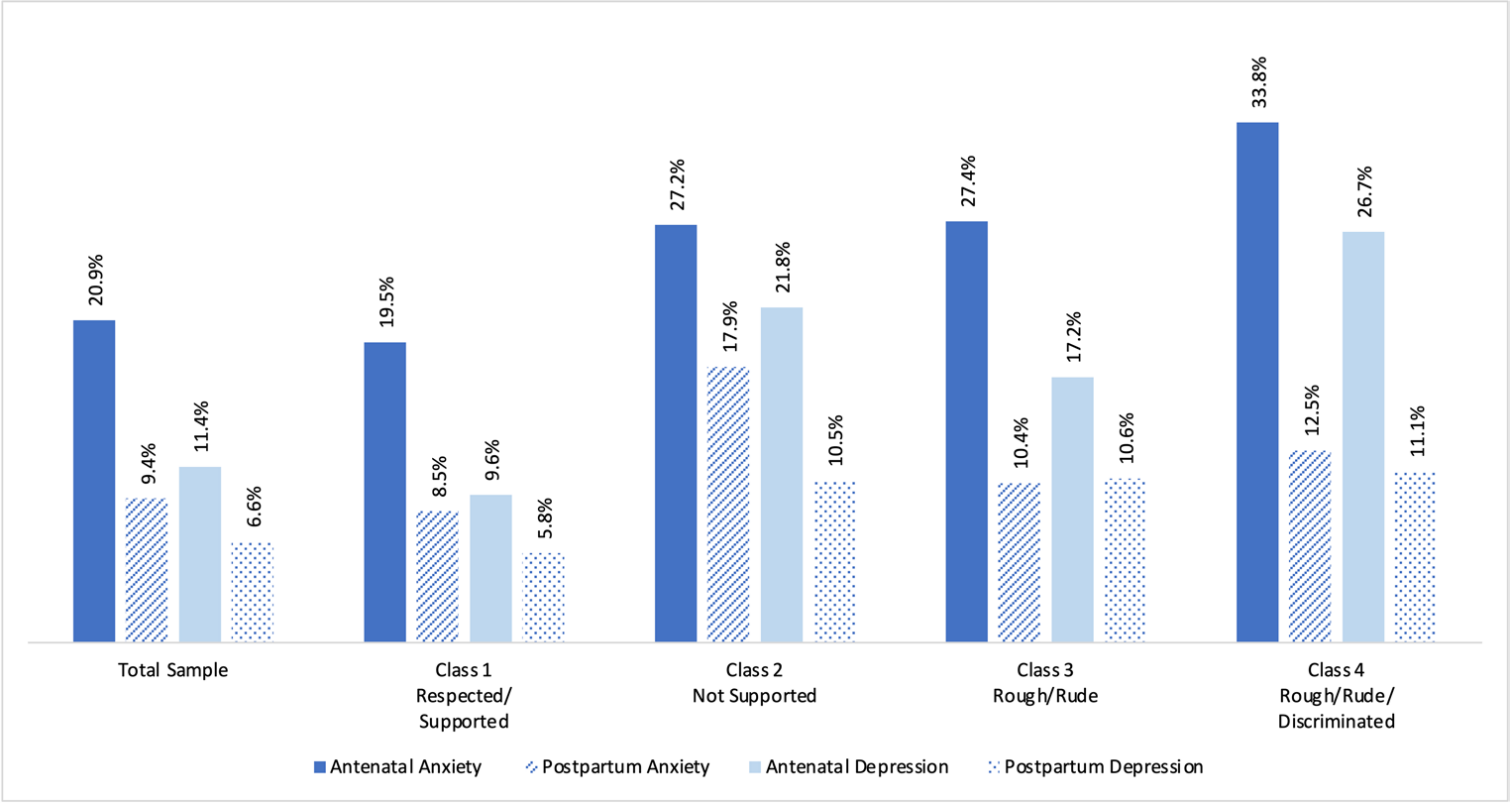
Percentages of respondents symptomatic for anxiety and depression by timeframe and class

Symptoms of anxiety and depression were reported by higher proportions of women during the antenatal period compared to the postpartum period (Fig. 4) within the total sample and across classes. Differences between antenatal and postpartum symptomatology were statistically significant for each class.

### Mental health symptoms differed across classes

When comparing proportions of women reporting symptomatology across the four classes, significant differences were observed for antenatal anxiety (*p* = 0.001), postpartum anxiety (*p* = 0.002), antenatal depression (*p* < 0.0001), and postpartum depression (*p* = 0.01; Fig. 4). Members of Class 1 (Respected/Supported) had the lowest proportion of women reporting perinatal mental health symptoms in both the antenatal period (anxiety 19.5%, depression 9.6%) and the postpartum period (anxiety 8.5%, depression 5.8%).

### Antenatal mental health was associated with reports of mistreatment

**Fig. 5:**
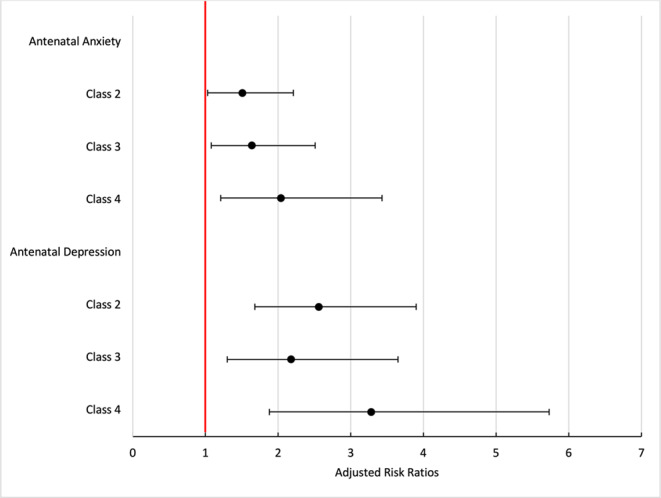
Adjusted relative risk of class membership by symptomatology for antenatal anxiety or depression. Covariates included age, parity, and doula support

Multinomial regression revealed that being symptomatic for antenatal anxiety or antenatal depression was associated with membership in classes categorized by mistreatment and/or lack of support (Table 2; Fig. 5). Effect sizes were higher for symptoms of antenatal depression versus symptoms of antenatal anxiety. Importantly, women who were symptomatic for antenatal depression (PHQ-2 ≥ 3) were 3.28 times at risk of being classified in Class 4 (Rough/Rude/Discriminated) versus Class 1 (Respected/Supported) in comparison to those who were not symptomatic for antenatal depression (PHQ-2 < 3), adjusting for age, parity, and presence of a doula (aRRR 3.28, CI95% 1.88–5.73). Additionally, women who reported antenatal GAD-2 ≥ 3 were 2.04 times at risk of being classified in Class 4 (Rough/Rude/Discriminated) versus Class 1 (Respected/Supported) in comparison to those with a GAD-2 < 3, adjusting for age, parity, and presence of a doula (aRRR 2.04, CI95% 1.21–3.43).

**Table 1:** Characteristics of respondents by class membership (*N* = 2539)

Characteristic	Total Sample, % (CI)	Class 1 Respected/Supported (*n* = 2146)^a^ % (CI)	Class 2 Not Supported (*n* = 176)^a^ % (CI)	Class 3 Rough/Rude (*n* = 131)^a^ % (CI)	Class 4 Rough/Rude/Discriminated (*n* = 86)^a^ % (CI)	*p*-value
Age (years) (mean/SD)	29.61 (0.12)	29.58 (0.13)	29.66 (0.47)	30.06 (0.55)	29.33 (0.70)	0.83
Race and ethnicity						< 0.0001
Asian/Pacific Islander	15.51 (13.98, 17.17)	15.07 (13.44, 16.86)	8.60 (5.02, 14.35)	19.93 (13.27, 28.81)	33.32 (23.01, 45.51)	
Black	4.60 (4.23, 5.00)	4.29 (3.86, 4.76)	6.39 (4.17, 9.66)	4.94 (2.61, 9.18)	8.20 (4.17, 15.51)	
Hispanic or Latina	50.07 (48.03, 52.11)	50.40 (48.17, 52.62)	49.79 (42.0, 57.60)	41.57 (32.98, 50.71)	56.08 (44.44, 67.09)	
Multiple or other races	3.21 (2.59, 3.97)	3.23 (2.56, 4.07)	6.01 (3.28, 10.76)	1.29 (3.00, 5.41)	-	
White	26.61 (24.8, 28.51)	27.01 (25.03, 29.10)	29.20 (22.42, 37.06)	32.26 (24.29, 41.43)	2.40 (0.54, 10.05)	
Language						< 0.0001
English	57.07 (55.03, 59.09)	57.43 (55.20, 59.62)	64.65 (56.73, 71.84)	62.62 (53.47, 70.95)	24.58 (16.20, 35.45)	
Spanish	16.79 (15.36, 18.33)	16.70 (15.15, 18.37)	11.26 (7.22, 17.15)	13.10 (8.22, 20.25)	35.83 (26.01, 47.00)	
English/Spanish equally	15.52 (14.10, 17.07)	15.83 (14.27, 17.52)	15.39 (10.50, 22.00)	14.43 (9.15, 22.03)	9.90 (4.99, 18.69)	
Other	10.61 (9.33, 12.05)	10.05 (8.70, 11.58)	8.70 (4.91, 14.96)	9.84 (5.43, 17.17)	29.69 (20.03, 41.59)	
Marital status						< 0.0001
Married or partnered	86.15 (84.71, 87.47)	86.83 (85.29, 88.24)	81.61 (74.84, 86.87)	91.19 (85.15, 94.92)	70.45 (59.57, 79.41)	
Single^b^	13.85 (12.53, 15.29)	13.17 (11.76, 14.71	18.39 (13.13, 25.16)	8.81 (5.08, 14.85)	29.55 (20.59, 40.43)	
Education						0.01
High school or less	32.90 (30.98, 34.89)	32.70 (30.62, 34.86)	30.69 (23.77, 38.61)	26.29 (19.10, 35.02)	53.19 (41.59, 64.46)	
Some college	32.97 (31.05, 34.95)	32.57 (30.49, 34.72)	38.80 (31.41, 46.74)	35.53 (27.28, 44.75)	27.43 (18.00, 39.42)	
Baccalaureate degree	18.73 (17.18, 20.39)	19.17 (17.48, 20.99)	14.22 (9.74, 20.29)	22.47 (15.87, 30.80)	10.45 (5.16, 20.00)	
Some grad school or more	15.39 (13.99, 16.91)	15.55 (14.03, 17.21)	16.29 (11.31, 22.90)	15.71 (10.16, 23.48)	8.93 (3.97, 18.86)	
Insurance						< 0.0001
Medi-Cal	48.62 (46.56, 50.67)	47.63 (45.40, 49.88)	55.98 (48.07, 63.59)	38.29 (30.01, 47.31)	74.79 (63.31, 83.62)	
Private	44.02 (41.98, 46.08)	45.0 (42.78, 47.25)	36.46 (29.25, 44.35)	55.79 (46.70, 64.51)	15.81 (8.79, 26.78)	
Other	7.37 (6.32, 8.56)	7.36 (6.23, 8.68)	7.56 (4.30, 12.97)	5.92 (2.79, 12.12)	9.40 (4.55, 18.40)	
Parity (mean/SD)	2.06 (0.03)	2.04 (0.03)	2.25 (0.13)	1.98 (0.12)	2.27 (0.14)	0.15
Gestation, weeks (mean/SD)	38.80 (0.04)	38.77 (0.04)	38.97 (0.14)	38.98 (0.13)	38.74 (0.23)	0.27
Doula present						0.08
No	83.63 (82.01, 85.14)	83.95 (82.18, 85.56)	82.43 (75.34, 87.81)	86.49 (79.24, 91.48)	72.80 (60.72, 82.24)	
Yes	16.37 (14.86, 17.99)	16.05 (14.44, 17.82)	17.57 (12.19, 24.66)	13.51 (8.52, 20.76)	27.20 (17.76, 39.28)	
Prenatal provider type						0.0001
Obstetrician/gynecologist	80.29 (78.68, 81.81)	81.38 (79.66, 82.99)	72.58 (65.33, 78.81)	78.38 (70.16, 84.83)	71.28 (60.32, 80.20)	
Family med/Other MD	5.89 (5.00, 6.93)	5.39 (4.48, 6.48)	6.62 (3.62, 11.78)	5.28 (2.32, 11.55)	18.03 (10.91, 28.32)	
Certified nurse midwife	7.37 (6.47, 8.38)	6.91 (5.96, 8.00)	12.96 (8.83, 18.62)	9.86 (5.79, 16.29)	3.63 (1.23, 10.25)	
NP, PA, or other	6.45 (5.53, 7.51)	6.31 (5.33, 7.46)	7.85 (4.59, 13.10)	6.48 (3.29, 12.38)	7.06 (3.29, 14.50)	
Prenatal provider choice						< 0.0001
Yes	79.96 (78.30, 81.53)	80.54 (78.74, 82.21)	74.34 (67.07, 80.47)	81.61 (73.54, 87.63)	74.08 (63.25, 82.60)	
No	19.28 (17.74, 20.92)	18.93 (17.27, 20.71)	24.58 (18.59, 31.76)	18.39 (12.37, 26.46)	19.11 (11.96, 29.11)	
No prenatal care	0.76 (0.47, 1.22)	0.54 (0.29, 0.98)	1.08 (0.22, 5.02)	-	6.81 (2.80, 15.66)	
Delivery provider type						0.01
Obstetrician/gynecologist	72.51 (70.83, 74.14)	72.78 (70.95, 74.54)	68.41 (60.87, 75.10)	80.28 (72.41, 86.33)	61.02 (49.41, 71.50)	
Fam medicine/Other MD	14.09 (12.72, 15.57)	13.77 (12.31, 15.36)	16.49 (11.37, 23.32)	11.5 (6.80, 18.79)	21.8 (13.64, 32.98)	
Certified nurse midwife	9.13 (8.37, 9.94)	9.39 (8.54, 10.32)	7.32 (4.44, 11.84)	7.47 (4.28, 12.72)	8.67 (4.43, 16.25)	
NP, PA, or other	4.27 (3.55, 5.13)	4.06 (3.30, 4.98)	7.77 (4.63, 12.75)	0.76 (0.11, 5.17)	8.52 (4.03, 17.10)	
Induction of labor						0.002
No	60.41 (58.39, 62.41)	61.96 (59.77, 64.10)	48.95 (41.26, 56.69)	57.99 (48.86, 66.61)	48.35 (37.27, 59.59)	
Yes	39.59 (37.59, 41.61)	38.04 (35.90, 40.23)	51.05 (43.31, 58.74)	42.01 (33.39, 51.14)	51.65 (40.41, 62.73)	
Pressured to be induced						< 0.0001
No	86.03 (84.55, 87.39)	88.63 (87.14, 89.97)	63.58 (55.83, 70.69)	86.42 (78.91, 91.55)	63.74 (51.90, 74.11)	
Yes	13.97 (12.61, 15.45)	11.37 (10.03, 12.86)	36.42 (29.31, 44.17)	13.58 (8.45, 21.09)	36.26 (25.89, 48.10)	
Epidural analgesia						< 0.0001
No	25.12 (23.40, 26.92)	24.41 (22.57, 26.36)	41.03 (33.63, 48.85)	18.38 (12.48, 26.22)	21.90 (13.96, 32.64)	
Yes	74.88 (73.08, 76.60)	75.59 (73.64, 77.43)	58.97 (51.15, 66.37)	81.62 (73.78, 87.52)	78.10 (67.36, 86.04)	
Pressured to have epidural						< 0.0001
No	88.87 (87.51, 90.11)	90.70 (89.31, 91.93)	77.17 (70.10, 82.97)	88.42 (81.22, 93.09)	66.17 (54.45, 76.18)	
Yes	11.13 (9.89, 12.49)	9.30 (8.07, 10.69)	22.83 (17.03, 29.90)	11.58 (6.91, 18.78)	33.83 (23.82, 45.55)	
Birth mode						0.27
Cesarean	30.50 (28.66, 32.40)	30.90 (28.89, 32.99)	23.6 (17.76, 30.64)	32.96 (24.97, 42.07)	30.37 (21.16, 41.48)	
Vaginal	69.50 (67.60, 71.34)	69.10 (67.01, 71.11)	76.40 (69.36, 82.24)	67.04 (57.93, 75.03)	69.63 (58.52, 78.84)	
Pressured to have cesarean						< 0.0001
No	88.77 (87.39, 90.01)	90.30 (88.87, 91.55)	78.78 (71.93, 84.32)	88.71 (81.79, 93.21)	69.43 (57.22, 79.41)	
Yes	11.23 (9.99, 12.61)	9.70 (8.45, 11.13)	21.22 (15.68, 28.07)	11.29 (6.79, 18.21)	30.57 (20.59, 42.78)	
Duration of labor, hours (mean, SD)	13.14 (29.05)	13.10 (0.32)	14.66 (1.27)	12.90 (0.98)	11.12 (1.41)	0.32
Admission to the NICU						0.04
No	86.45 (84.97, 87.81)	87.24 (85.67, 88.67)	82.87 (76.04, 88.06)	83.75 (75.75, 89.48)	76.99 (65.29, 85.61)	
Yes	13.55 (12.19, 15.03)	12.76 (11.33, 14.33)	17.13 (11.94, 23.96)	16.25 (10.52, 24.25)	23.01 (14.39, 34.71)	

CI = 95% Confidence Interval, LOS = length of stay, MD = medical doctor, NP = nurse practitioner, PA = physician assistant

^a^Ns reflect unweighted counts and %s reflect survey-weighted proportions

^b^Single, never married or separated, divorced, or widowed

## Postpartum mental health was associated with membership in class 2 (not supported)

**Fig. 6:**
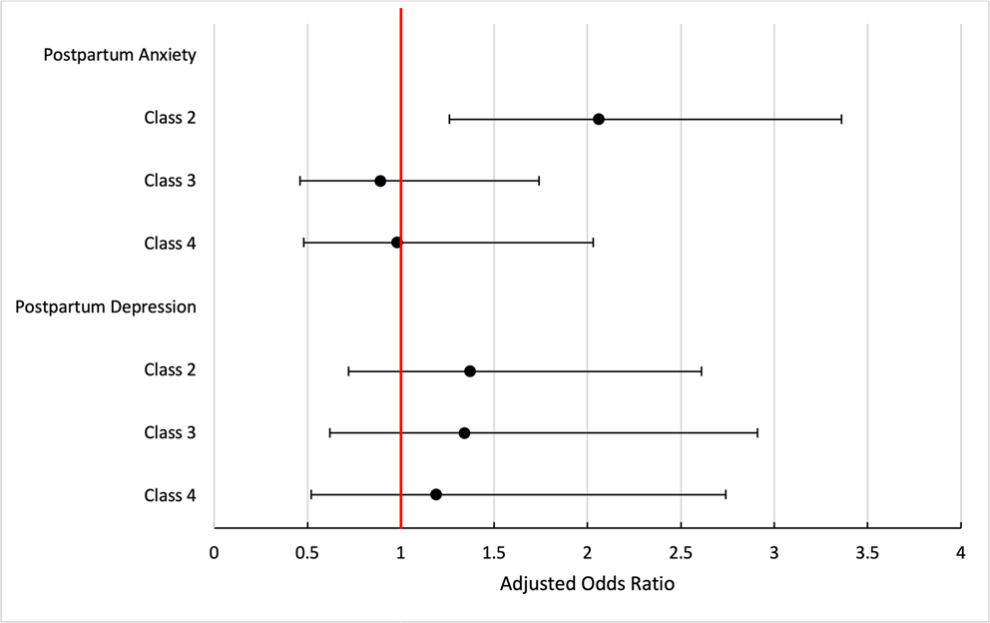
Adjusted risk of symptomatology for postpartum anxiety or depression by class membership. Covariates included age, parity, doula support, antenatal anxiety, and antenatal depression

Logistic regression revealed only one statistically significant relationship when examining postpartum mental health by class membership (Table 2; Fig. 6). Compared to members of Class 1 (Respected/Supported), those who were members of Class 2 (Not Supported) had 2.06 times higher risk of being symptomatic for postpartum anxiety (GAD-2 ≥ 3), adjusting for age, parity, and presence of a doula (aRRR 2.06, CI95% 1.26–3.36).

**Table 2:** Unadjusted and adjusted associations between class membership and symptomatology for anxiety and depression

	Multinomial Logistic Regressions				Logistic Regressions				
	Antenatal Anxiety		Antenatal Depression		Postpartum Anxiety			Postpartum Depression	
	RRR (95%CI)	aRRR (95%CI)	RRR (95%CI)	aRRR (95%CI)	OR (95%CI)		aOR (95%CI)	OR (95%CI)	aOR (95%CI)
Class 1 - Respected/Supported	Ref	Ref	Ref	Ref	Ref		Ref	Ref	Ref
Class 2 - Not Supported	**1.54 (1.06–2.24)**	**1.51 (1.03–2.21)**	**2.63 (1.74–3.98)**	**2.56 (1.68–3.90)**	**2.34 (1.48–3.70)**		**2.06 (1.26–3.36)**	**1.89 (1.07–3.36)**	1.37 (0.72–2.61)
Class 3 - Rough/rude	**1.55 (1.02–2.38)**	**1.64 (1.08–2.51)**	**1.96 (1.18–3.24)**	**2.18 (1.30–3.65)**	1.24 (0.66–2.35)		0.89 (0.46–1.74)	**1.92 (1.02–3.62)**	1.34 (0.62–2.91)
Class 4 - Rough/Rude/Discriminatory	**2.11 (1.29–3.44)**	**2.04 (1.21–3.43)**	**3.44 (2.03–5.80)**	**3.28 (1.88–5.73)**	1.53 (0.76–3.08)		0.98 (0.48–2.03)	2.03 (1.00–4.09.00.09)	1.19 (0.52–2.74)

Adjusted multinomial logistic regressions include these covariates: age, parity, doula support

Adjusted logistic regressions include these covariates: age, parity, doula support, antenatal anxiety, antenatal depression

## Discussion

We identified four distinct latent classes of respectful care and mistreatment. Reassuringly, the latent class with the largest sample size was characterized by respectful and supportive care. A concerning result was that about one in six women were members of classes characterized by lack of support or mistreatment.

Examining the characteristics of classes categorized by lack of support or mistreatment may provide insights about populations most vulnerable to these types of treatment. Although their demographic and socioeconomic characteristics differed, Class 2 and Class 4 had high proportions of women who reported pressure to receive childbirth interventions. Class 2 also had the highest proportion of women with graduate or higher education, which is consistent with evidence that women with a higher level of education had lower risk of reporting an optimal birth experience (van der Pijl et al. 2021). By contrast, members of Class 4 may have been more vulnerable to mistreatment due to their demographic and socioeconomic characteristics (i.e., more respondents who were people of color, had lower education, had public health insurance) compared to other classes. Notably, almost 7% of members of Class 4 did not receive prenatal care compared to 1% or fewer in other classes. These results, in combination with international findings that mistreatment was associated with postpartum depression in both private and public hospitals (Leite et al. 2020), suggest that future research is needed to examine the roles of income and insurance status as potential mechanisms that may explain the relationship between mistreatment and mental health. Additionally, research examining the Listening to Mothers dataset, in which continuous social support was associated with a 50% reduced prevalence of perinatal mood and anxiety disorder symptomatology (Feinberg et al. 2022), suggests that continuous social support may offer a modifiable factor to reduce perinatal mental health symptomatology.

Multiple demographic and socioeconomic factors have been identified as risk factors for mistreatment during maternity care and childbirth (Bohren et al. 2019; Liu et al. 2024; Mohamoud et al. 2023); yet, little evidence has examined antenatal anxiety and antenatal depression as risk factors (Guure et al. 2023). An important contribution of this study is the identification of symptoms of antenatal anxiety and antenatal depression as risk factors for mistreatment during childbirth. We observed 2 to 3 three times higher risk of symptoms of antenatal depression and 1.5 to 2 times higher risk of symptoms of antenatal anxiety among women who were members of classes characterized by mistreatment or lack of support.

An unexpected finding was that most classes characterized by mistreatment did not have increased risk of symptoms of postpartum anxiety or postpartum depression. Notably, members of Class 4 (characterized by rough, rude, and discriminatory treatment) were more often women of color who did not speak English, had less education, and had public health insurance. Evidence suggests that women of color may be less likely to disclose symptoms of mental illness due to cultural stigma, distrust of healthcare providers, and fear of child protective services involvement (Beck 2023), which could explain the lower risk of reported symptoms of postpartum anxiety or postpartum depression among members of Class 4.

Our results highlight the importance of providing respectful, patient-centered care for women who are experiencing perinatal mental health conditions. Routine screening must occur during prenatal care to identify women with diagnoses of antenatal anxiety and depression. Maternity care clinicians must be educated and trained to provide appropriate care to women with perinatal mental health conditions. Pregnant women with mental health conditions must also receive education and resources to prepare them for the physical and mental challenges inherent in childbirth and the postpartum period.

Some limitations of this study should be noted. The cross-sectional design of the original survey does not permit an analysis of mistreatment over time during the hospital stay (i.e., upon admission, during labor and birth, during the postpartum period). Moreover, the retrospective design of the original survey may have resulted in recall bias. A related limitation of the retrospective design is the use of the PHQ-4, which is a very brief screening tool, to retrospectively measure symptoms of anxiety and depression during pregnancy. Results describing antenatal depression using the PHQ-4 in this sample have been published elsewhere (Declercq et al. 2022). Another limitation was the lack variables identifying specific perpetrators of mistreatment and frequency of mistreatment. There was no information in the dataset about diagnoses of chronic anxiety or depression, which did not permit inclusion of these variables in our analyses.

An additional limitation of the study was that half of the sample was Hispanic, reflecting the birthing population of California in 2016, which is not nationally representative of the U.S population. A phenomenon noted in healthcare literature as the Hispanic paradox refers to the tendency of Hispanic Americans to have better health outcomes when compared to non-Hispanic Americans, possibly due to strong family and social connections (Brown et al. 2007). Thus, our sample may have under-represented mistreatment during childbirth and symptoms of perinatal mental health conditions. Social desirability bias is another limitation that may have resulted in lower reports of mistreatment and symptoms of perinatal mental health symptoms than experienced by respondents.

## Conclusions

In this study we identified that women who were symptomatic for antenatal anxiety or antenatal depression were at increased risk for mistreatment and/or not receiving support during childbirth. Women who did not feel supported during childbirth had increased risk of being symptomatic for postpartum anxiety. Results suggest a need to provide additional support for women who are diagnosed with antenatal anxiety and/or depression and to educate maternity care clinicians about potential effects of mistreatment during childbirth on postpartum mental health.

## Supplementary Information

Below is the link to the electronic supplementary material.

Supplementary File 1 (DOCX 15.2 KB)

## Data Availability

The dataset supporting the conclusions of this article is available in the Odum Institute Dataverse public data repository at the University of North Carolina, (https://dataverse.unc.edu/dataset.xhtml?persistentId=doi:10.15139/S3/3KW1DB).
