# Supplementary material for: Patterns of respectful care and mistreatment during childbirth in relation to perinatal mental health: a secondary analysis of listening to mothers in California
Source: Arch Womens Ment Health. 2026 Mar 7;29(2):46. doi: 10.1007/s00737-025-01656-0 (PMC12966254; doi:10.1007/s00737-025-01656-0)
Supplement: Supplementary file 1 — (DOCX 15.2 KB) [file 737_2025_1656_MOESM1_ESM.docx]

**Title:** Patterns of Respectful Care and Mistreatment During Childbirth in Relation to Perinatal Mental Health: A Secondary Analysis of Listening to Mothers in California

**Authors:** Stacey E. Iobst^1^ & Elise N. Erickson^2^

^1^Corresponding author; Towson University, College of Health Professions, Department of Nursing, 8000 York Road, Towson, Maryland 21252, [siobst@towson.edu](mailto:siobst@towson.edu)

^2^University of Arizona, College of Medicine, Department of Physiology, 1656 E Mabel Street, Rm 424, P.O. Box 245051, Tucson, AZ 85724

**Supplemental Table 1.** Latent class model fit statistics

|  | LL | AIC | BIC | VLMR | VLMR  p-value | Entropy | Smallest Class Count (n) | Smallest Class Size (%) |
| --- | --- | --- | --- | --- | --- | --- | --- | --- |
| 2-Class | -5148.55 | 10337.10 | 10453.89 | 1285.67 | 0 | 0.7023 | 449 | 17.67 |
| 3- Class | -4980.52 | 10019.04 | 10188.39 | 336.06 | 0 | 0.7277 | 218 | 8.59 |
| 4- Class | -4898.02 | 9872.03 | 10093.93 | 165.01 | 0 | 0.7959 | 86 | 3.39 |
| 5- Class | -4841.18 | 9776.37 | 10050.82 | 113.67 | 0 | 0.7426 | 44 | 1.75 |
| 6- Class | -4830.77 | 9773.55 | 10100.56 | 20.82 | 0.06 | 0.7236 | 45 | 1.77 |
| 7- Class | -4811.39 | 9752.78 | 10132.35 | 38.77 | 0.002 | 0.6913 | 30 | 1.20 |
| LL=log-likelihood, ﻿AIC = Akaike information criterion, BIC = Bayesian information criterion, ﻿VLMR-LRT = Vuong-Lo-Mendell-Rubin test | | | | | | | | |
